# Emergence of Different Gaits in Infancy: Relationship Between Developing Neural Circuitries and Changing Biomechanics

**DOI:** 10.3389/fbioe.2020.00473

**Published:** 2020-05-19

**Authors:** Arthur Henri Dewolf, Francesca Sylos-Labini, Germana Cappellini, Francesco Lacquaniti, Yury Ivanenko

**Affiliations:** ^1^Department of Systems Medicine and Center of Space Biomedicine, University of Rome Tor Vergata, Rome, Italy; ^2^Laboratory of Neuromotor Physiology, IRCCS Santa Lucia Foundation, Rome, Italy; ^3^Department of Pediatric Neurorehabilitation, IRCCS Santa Lucia Foundation, Rome, Italy

**Keywords:** early development, human bipedal locomotion, gait transitions, biomechanical gait determinants, neural control of different gaits, infants

## Abstract

How does gait-specific pattern generation evolve in early infancy? The idea that neural and biomechanical mechanisms underlying mature walking and running differ to some extent and involve distinct spinal and supraspinal neural circuits is supported by various studies. Here we consider the issue of human gaits from the developmental point of view, from neonate stepping to adult mature gaits. While differentiating features of the walk and run are clearly distinct in adults, the gradual and progressive developmental bifurcation between the different gaits suggests considerable sharing of circuitry. Gaits development and their biomechanical determinants also depend on maturation of the musculoskeletal system. This review outlines the possible overlap in the neural and biomechanical control of walking and running in infancy, supporting the idea that gaits may be built starting from common, likely phylogenetically conserved elements. Bridging connections between movement mechanics and neural control of locomotion could have profound clinical implications for technological solutions to understand better locomotor development and to diagnose early motor deficits. We also consider the neuromuscular maturation time frame of gaits resulting from active practice of locomotion, underlying plasticity of development.

## Introduction

What are the general characteristics of maturation of gait-specific pattern generation circuitries? Even though humans start to walk significantly later than most animals (Garwicz et al., 2009), stepping-like responses can be evoked in human neonates. These steps are very irregular and typically disappear few weeks after birth and reappear later when they evolve into intentional locomotion (Forssberg, 1985; Thelen and Cooke, 1987). Recent advances in biotechnology along with reduced physical size of electromechanical systems has enabled to unveil new information about the locomotor output of the stepping behavior (Zhu et al., 2015; Redd et al., 2019; Airaksinen et al., 2020). By comparing this stepping behavior with adult walking, it has been shown that the primitive muscular control patterns observed in neonates are highly preserved and recombined during development, supporting the idea that this early stepping is a precursor to adult walking (Dominici et al., 2011; Sylos-Labini, La Scaleia, Cappellini, Fabiano, Picone, Keshishian, 2020), in spite of noticeable differences with mature gait (Forssberg, 1985; Ivanenko et al., 2013a; Yang et al., 2015). The infants also show the elements of quadrupedal coordination during stepping (La Scaleia et al., 2018), swimming (McGraw, 1939), or crawling (Patrick et al., 2012; Forma et al., 2019). However, the developmental path from the neonate behaviors to adult running gaits is still unknown.

While the specific features of the walk and run are clearly distinct in adults, there is little evidence for such distinct walking and running patterns at the onset of independent locomotion. Instead, the characteristics of gaits show gradual and progressive values during growth (Whitall and Getchell, 1995). In other words, the locomotor patterns in young children do not fall nicely into a classic category like walking or running (Vasudevan et al., 2016), but such distinction is stretched out over developmental time. Here, we argue that these two different modes of locomotion most likely evolved from similar circuitry, and represent a specific kind of developmental bifurcation with different maturational rates.

In the first sections, we briefly highlight the main features of the two modes of mature human locomotion and neurophysiological and biomechanical considerations for the control of different animal gaits. Next, we consider recent findings on the process of development of neural network, and the absence of clear distinction in infant stepping. In a final section, we discuss common elements of organization with different modes of locomotion, and how early motor experience during development may shape motor properties in different environmental contexts including early gait impairments in infancy.

## The Two Modes of Human Locomotion

**Figure 1:**
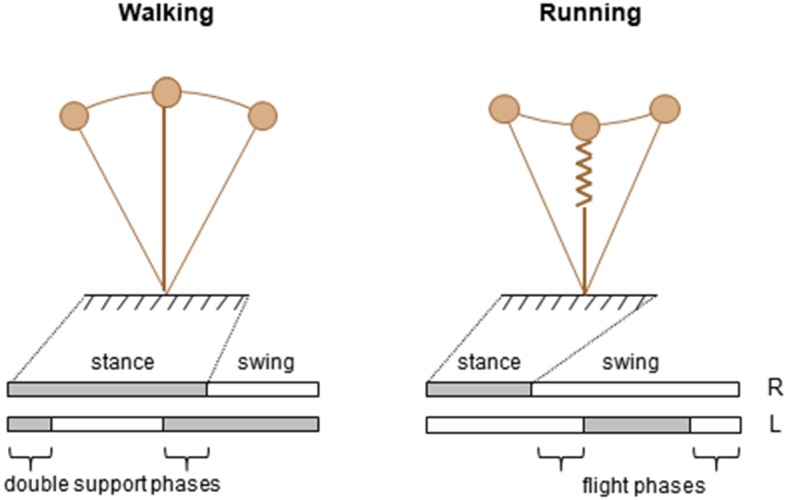
Schematic representation of two modes of human locomotion during stance phase.

Among a vast variety of possible ways of locomotion, humans generally prefer just two, categorized into walking and running (Cavagna et al., 1988). Mature walking gait can be caricatured by the hip joint swinging over a relatively straight leg, whereas mature running gait can be seen as a bounce off compliant leg followed by parabolic flight (Figure 1). Few variables clearly distinguish features of walking and running gait and represent the essence of these commonly produced behavioral patterns (called *collective variables*). Once identified, the process that underlies changes in locomotor behavior across the lifespan may be studied.

One key parameter to discriminate forms of locomotion has traditionally been the difference observed in the spatiotemporal features, specifically the relative timing of the feet contacts with the ground (Figure 1, lower panels). Indeed, periods of single support intersperse with periods of double support during walking and with periods of flights during running. Accordingly, walking and running are operationally distinguished based on the presence (running) or absence (walking) of an aerial phase during which neither foot is in contact with the ground. Therefore, the existence or absence of a flight phase is one well-accepted collective variable.

Another distinguishing feature between adult walking and running is the path of the center of mass (COM) of the body (Figure 1, upper panels). The basis for this approach is the work of Cavagna and co-workers (Cavagna et al., 1988), who showed that major characteristics that serve as signatures for human running and walking are the interaction between the forward and the vertical displacement of the center of mass of the body (COM). In walking, the COM reaches its lowest point when its forward velocity is maximal; this behavior characterizes a pendulum-like energy exchange between potential and kinetic energy. In running, the COM reaches its lowest point when its forward velocity is minimal; this behavior characterizes a storage-release of elastic energy. These mechanisms were defined as the “inverted pendulum” mechanism in walking, and the “pogo-stick bouncing” in running (Figure 1).

The trajectory of the COM in space in turn depends on the combined rotation of lower-limb segments (Lacquaniti et al., 1999, 2002; Dewolf et al., 2018). During both running and walking, the behavior of the COM is the result of a gait-dependent control of phase relationship between the lower-limb segments (Kao et al., 2000; Ivanenko et al., 2007a), also a distinguishing feature of gaits.

## Neurophysiological Considerations for The Control of Different Gaits

It is known that such gait coordination results from interplay between the activity of spinal central pattern generators (CPGs), sensory signals originating in the limbs and supraspinal signals (Grillner, 1981; Büschges et al., 1995). There are at least two conceptual models on how the locomotor circuitry may be organized (for a more comprehensive overview of hypotheses on CPG organization, see, for example, Duysens et al., 2013; Rybak et al., 2015; Kiehn, 2016; Minassian et al., 2017; Grillner and El Manira, 2020). One model considers a set of unit CPGs controlling specific groups of muscles (Grillner and El Manira, 2020). Another model consists of a two-layered CPG organization with one rhythm-generating circuit and another one for downstream control of muscle activity and coordination of different gaits (walk, trot, and gallop) (McCrea and Rybak, 2008; Danner et al., 2017).

**Figure 2:**
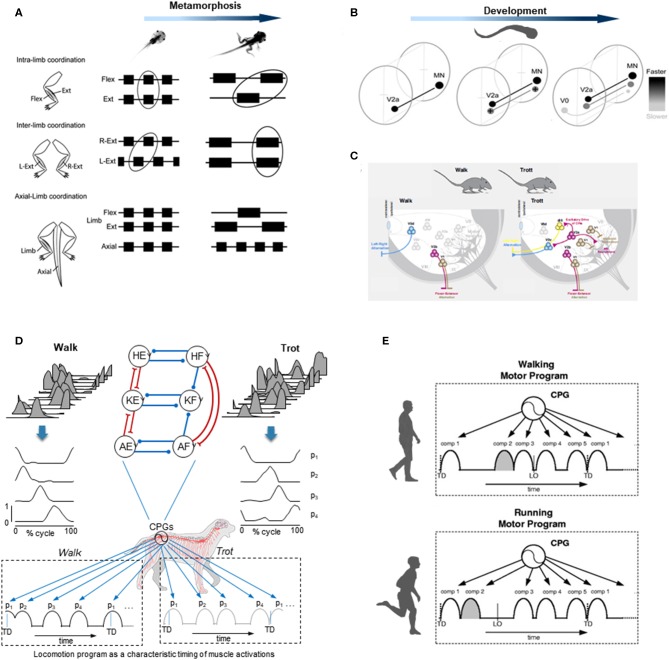
Examples of gait-specific changes in spinal locomotor controllers. **(A)** changes in coordination between extensor, flexor, and axial muscle bursts during Xenopus metamorphosis (modified from Rauscent et al., 2006 with permission from Elsevier). **(B)** schematic summary of changes in the development and recruitment of spinal circuitry in larval Zebrafish: neurons responsible for progressively slower movements in larvae are added as zebrafish develop (modified from Fetcho and McLean, 2010 with permission). **(C)** distinct spinal interneurons circuits drive different gaits in mice (modified from Deska-Gauthier and Zhang, 2019 with permission from Elsevier). **(D)** locomotion program as a characteristic timing of muscle activations during walk (*left*) and trot (*right*) in dogs. *From top to bottom:* averaged hindlimb EMG data, basic activation patterns (p1-p4) obtained by decomposing muscle activity and a schematic sequence of activation patterns for each gait (modified from Catavitello et al., 2015). Schematic representation of the unit burst generator CPG model is also plotted on the top (circles are interneurons controlling hip (H), knee (K), and ankle (A) extensors (E) and flexors (F), excitatory and inhibitory connections are represented by lines ending with forks or circles, respectively) (redrawn from Grillner and El Manira, 2020). **(E)** locomotion motor program as a sequence of activation pulses for walking and running in humans (modified from Cappellini et al., 2006).

Several studies on animals have shown that the CPG circuits reside mainly in the ventral aspect of the spinal cord (Kiehn, 2016; Grillner and El Manira, 2020), and are involved in changing the mode of locomotion. While the intensity of supraspinal inputs may determine the speed and mode of locomotion, the spinal circuitry is able to implement specific coordination patterns for different gaits. A classical physiological study on decerebrated cat stepping on a treadmill showed that increasing the strength of electrical stimulation of the mesencephalic locomotor region can make the gait changes from a slow walk to trot and finally gallop (Shik et al., 1966; Shik and Orlovsky, 1976). Another example of gait-related spinal control can be obtained during fictive swimming in the lamprey: varying the concentration of neurotransmitter applied to the lamprey spinal cord produces changes in the intersegmental coordination (Matsushima and Grillner, 1992). In humans, by using mental imagery of locomotion in fMRI, Jahn et al. (2008) have suggested that the supraspinal network of quadrupeds is conserved in both walking and running gaits, despite the transition to bipedalism. The similarities of the basic organization of supraspinal locomotor control for gait and speed regulation in humans and cats (Drew et al., 2004) suggest similar gait-related spinal circuitries across mammals. In sum, it is worth stressing that the neural mechanisms for the control of different gaits involve both shared and specific neural circuits. Figure 2 illustrates examples of such gait-dependent changes in spinal locomotor controllers.

Using electrophysiological, pharmacological, or neuroanatomical approaches in invertebrates and vertebrates, the identification of the spinal interneurons and investigation of the locomotor output provided important insights into the gait-dependent organization of CPGs and how these functional circuits are formed during development. An example of developmental process can be observed during the Xenopus metamorphosis (Figure 2A). Limb and tail muscle coordination switches from pro-metamorphosis to metamorphic climax, suggesting that the neural network is progressively reshaped to allow the transformation from aquatic swimming to ground stepping (Rauscent et al., 2006). Such plasticity results from both a dynamic reconfiguration of spinal circuitry and the involvement of new circuitry (Combes et al., 2004). Studies focused on the patterns of recruitment of interneurons in the spinal motor system of zebrafish led to principles underlying the reorganization of spinal circuitry (Fetcho and McLean, 2010). The neurons producing fast movements are established early and, as the zebrafish develops, interneurons responsible for low frequency movements are progressively added (Figure 2B). At the end of development, the neurons producing slow, intermediate, or fast movements can be recruited either separately or combined sequentially to increase the locomotor speed (McLean et al., 2008; Grillner and El Manira, 2020). Studies on gait-related spinal circuits in mice demonstrated intriguing similarities with the zebrafish spinal cord (McLean et al., 2008; Fetcho and McLean, 2010). For example, in both species, V1 interneurons are critical for setting the speed of locomotion, and sequential V2a recruitment is observed with increasing speed (Ausborn et al., 2012). However, a major difference is that as they speed up, mice (as the great majority of terrestrial quadrupedal mammals) change their gait from walk to trot and to gallop (Figure 2C), and the inter-limb coordination switches from alternation during both walking and trotting to synchrony during galloping. Such gait-dependent left–right rhythmicity and coordination recruitments are mediated by speed-dependent spinal interneurons (Figure 2C). According to the unit-burst generator organization, spinal interneurons coordinate the activity of “excitatory core burst generators” dedicated to coordination of hip, knee and ankle flexor, and extensor motor output of each limb (Grillner and Jessell, 2009; Grillner and El Manira, 2020), which can be combined to produce various gait patterns (Catavitello et al., 2015; Figure 2D). One alternative concept of the CPG organization includes a separation of the networks for rhythm generation and motoneuron excitation. According to this hypothesis, the rhythm generating circuit would set the rhythm for one limb, and then interneurons would activate a certain set of motoneurons and inhibit others depending on gaits (Lafreniere-Roula and McCrea, 2005; Shevtsova and Rybak, 2016; Danner et al., 2017; Ausborn et al., 2018). Both approaches emphasize gait-specific coupling of elements of pattern generation circuitries, mediated by speed-dependent spinal interneurons.

Another way to get insight into CPG functioning for different gaits is to look at the spatiotemporal organization of the total locomotor output and multi-muscle activity patterns in particular. In dogs as in several other animal species including humans, muscle activity patterns (Figure 2D) can be decomposed into a set of four basic temporal patterns that account for ~90% of total variance (Dominici et al., 2011; Catavitello et al., 2015). These basic temporal patterns have specific timings during a gait cycle (Figure 2D), consistent with “drive pulse” rhythmic elements in the spinal circuitry of zebrafishes, frogs, or mice (Rauscent et al., 2006; Fetcho and McLean, 2010; Giszter et al., 2010). The specific timing of each basic temporal pattern differs between different gaits to produce various intra- and inter-limb coordination, as it does for human walking and running (Cappellini et al., 2006; Figure 2E). In both running and walking, the muscles activated by each basic temporal pattern are roughly similar, suggesting some degree of commonality (Cappellini et al., 2006). However, differences are also noticeable (Santuz et al., 2019), such as the number of modules that show mode-dependent modulation, with additional patterns detected during running as compared to walking (Yokoyama et al., 2016). Recent data from vertebrates indicates that the structure of the basic patterns extracted from EMGs may originate from spinal interneuronal networks (Caggiano et al., 2016; Takei et al., 2017). It is therefore plausible that the functioning of gait-related spinal circuits is reflected in the mode-dependent modulation of basic activation patterns. The mode-dependency in the neural networks underlying human locomotion is consistent with the speed control mechanism of vertebrate CPGs, providing indirect evidence for phylogenetically conserved neural circuits of locomotion (Grillner and Jessell, 2009; Yokoyama et al., 2016). The idea that neural mechanisms underlying walking and running are partly independent in adulthood is further supported by previous studies showing that newly acquired locomotor patterns at slow speed rarely transfer to fast speed movements (Vasudevan and Bastian, 2010; Ogawa et al., 2012, 2015). Taken together, these observations might reflect the fact that, even though there are shared neural circuits for different gaits, in adults there is no simple scaling of motoneuron and interneuron activity from walking to running, but the involvement of somewhat different neural circuits.

In addition to examples illustrated in Figure 2, there are also other important aspects related to the maturation rates of gait-specific pattern generation networks. There might be different rates of maturation in different animals; for instance, the development of spinal interneurons observed in zebrafish (Figure 2B) may not necessarily apply to other species. Nevertheless, increasing evidence suggests a similar developmental pattern of neurons in vertebrates (Cepeda-Nieto et al., 2005; Fetcho and McLean, 2010). Interestingly, it has been shown that walking and running in non-human bipeds do not mature at the same rate, with a running pattern relatively mature earlier in life in chicks (Muir et al., 1996). The fact that chicks can innately run almost as well as an adult may suggest that, as in zebrafish, the interneurons mainly involved in the production of fast movement are developed early. Humans have a relatively long period of gait development (Garwicz et al., 2009) and, in the following sections, we will specifically consider the organization and maturation of gait patterns in humans.

## General Features and Maturation of Gait Patterns from Neonate to Adult

**Figure 3:**
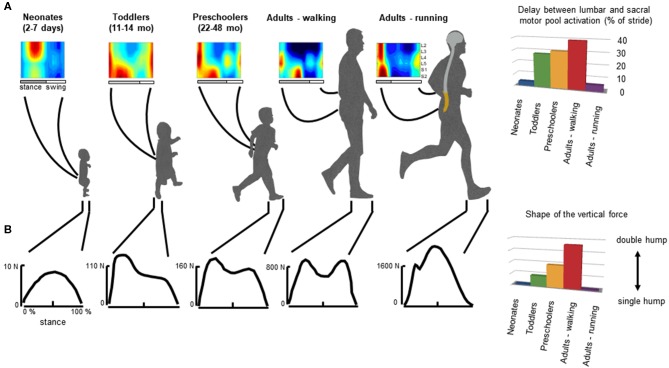
General features of gait patterns from neonate to adult. **(A)** spatiotemporal maps of motoneuron activity of the lumbosacral enlargement in neonates, toddlers, preschoolers, and adults walking and running, and the delay between the maximum activation of lumbar and sacral motor pools (data from Ivanenko et al., 2013a for stepping, and Cappellini et al., 2010 for running). **(B)** typical vertical loading force during stepping in neonates, toddlers, preschoolers, and adults walking and running, and the characteristic force profile evaluated using an adapted ratio of the coefficients of Fourier series (Hallemans et al., 2006a) providing a description of the main features of the shape of the ground reaction force: when the ratio is nil the force curve has a single hump whereas when the ratio increases, the force curve tends to have a double hump shape.

The CPGs in vertebrates emerged during evolution from a common ancestral circuit (Grillner and Jessell, 2009; Kiehn, 2016) and it has been suggested that, in humans, locomotor modules evolved from similar circuitry (Dominici et al., 2011; Yokoyama et al., 2016). In humans, when EMG activity patterns are mapped onto the spinal cord in approximate rostrocaudal locations of the motoneuron (MN) pools, the activation of MNs tends to occur in bursts (Figure 3) that can be associated with the major kinetic or kinematic events of the gait cycle in a gait-specific manner (Ivanenko et al., 2008a; Cappellini et al., 2010; La Scaleia et al., 2014; Yokoyama et al., 2017; Dewolf et al., 2019a). In particular, the sacral activation timing is clearly gait-dependent (Ivanenko et al., 2008a). It is worth stressing that these gait-specific features undergo functional reorganization during development from the neonate to the adult.

In adults, during walking the COM vaults over a relatively stiff limb with the heel well in front of the hip at the beginning of stance, and the heel lift with a maintained toe contact at the end of stance. One of the direct consequences of such heel-to-toe roll-over pattern is that the extension of distal joints is delayed relative to proximal joints, leading to the typical double-hump shape (so-called ≪*m*−*pattern*≫) of the vertical ground reaction force (Figure 3, bottom panel), characterized by Fourier analysis (based on the relationship between the shape of the force and the ratios of the coefficients of the Fourier series, Alexander and Jayes, 1980; Hallemans et al., 2006b). In addition, separate lumbar and sacral activations are observed: muscle activations intervene close to the apexes of the m-pattern to re-excite the inverted-pendulum oscillations of the system (Ivanenko et al., 2008a; Lacquaniti et al., 2012; Dewolf et al., 2019a). Conversely, during running with the center of mass rebounding on compliant spring legs, the vertical force exerted on the ground presents a single-hump shape (Nilsson and Thorstensson, 1989; Dewolf et al., 2016), and both the lumbar and sacral activations intervene close to the apexes of the ground reaction force (Ivanenko et al., 2008a; Cappellini et al., 2010; Yokoyama et al., 2017) (Figure 3).

**Figure 4:**
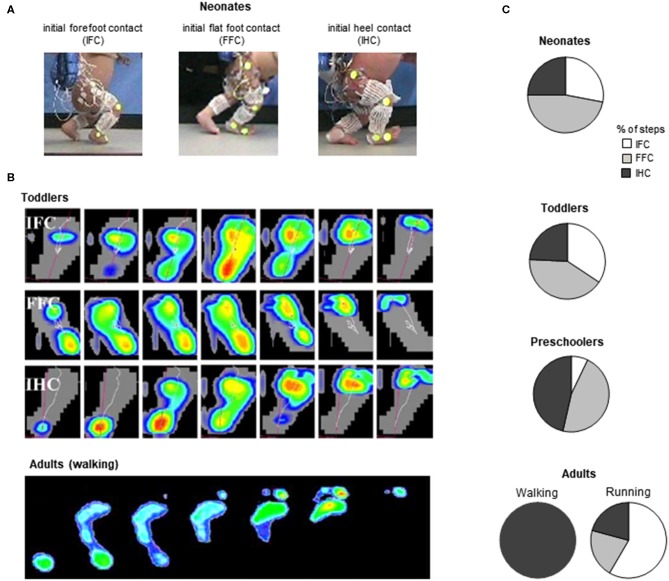
General foot placement characteristics during stepping. **(A)** examples of initial forefoot (IFC), flat foot (FFC), and heel (IHC) contacts in three neonates (adapted from Sylos-Labini et al., 2017). **(B)** from top to bottom: three different foot-contact patterns in toddlers (IFC, FFC, and IHC) and plantar pressure prints (left foot) of a typical adult “heel-to-toe” rolling pattern during the stance phase (redrawn from Hallemans et al., 2006b with permission from Elsevier). **(C)** percent of steps with different types of touchdown contacts for stepping in neonates (from Sylos-Labini et al., 2017), toddlers and preschoolers (from Hallemans et al., 2006b) and adult walking and running (from Larson, 2014).

In neonates, stepping lacks these specific features of adult heel-to-toe roll-over walking pattern (Forssberg, 1985; Dominici et al., 2011), and the foot placement characteristics in neonates showed wide variations (Figure 4A, Sylos-Labini et al., 2017). Three major footfall patterns were identified with the initial heel, midfoot, and forefoot contacts, respectively (Figure 4C). However, even when the neonates demonstrated a heel initial contact, the general features of gait patterns markedly differed relatively to adult. Indeed, the two peaks in the vertical ground reaction force and the associated MNs bursts were lacking. Instead, the vertical force exerted on the ground (neonates are generally able to support ~30% of their weight) presents a single-hump shape, even if some influences of the manual body weight support on the ground reaction force cannot be excluded (Forssberg, 1985; Sylos-Labini et al., 2017). However, when adults walk with a body weight support system, the kinetic events defining the “m-pattern” (i.e., the early stance peak of vertical force, the mid-stance interval, and the late stance peak) are recognizable in the force profiles up to 75% of body weight support (Ivanenko et al., 2002). In addition, during stance, antigravity leg muscles tend to be co-activated with a quasi-sinusoidal waveform, corresponding to the single-hump shape of the force, independently of the level of body weight support (Sylos-Labini, La Scaleia, Cappellini, Fabiano, Picone, Keshishian, 2020). In turn, a quasi-synchronous lumbar and sacral activations is observed (Figure 3A), corresponding to the single peak of vertical force (Ivanenko et al., 2013a).

**Figure 5:**
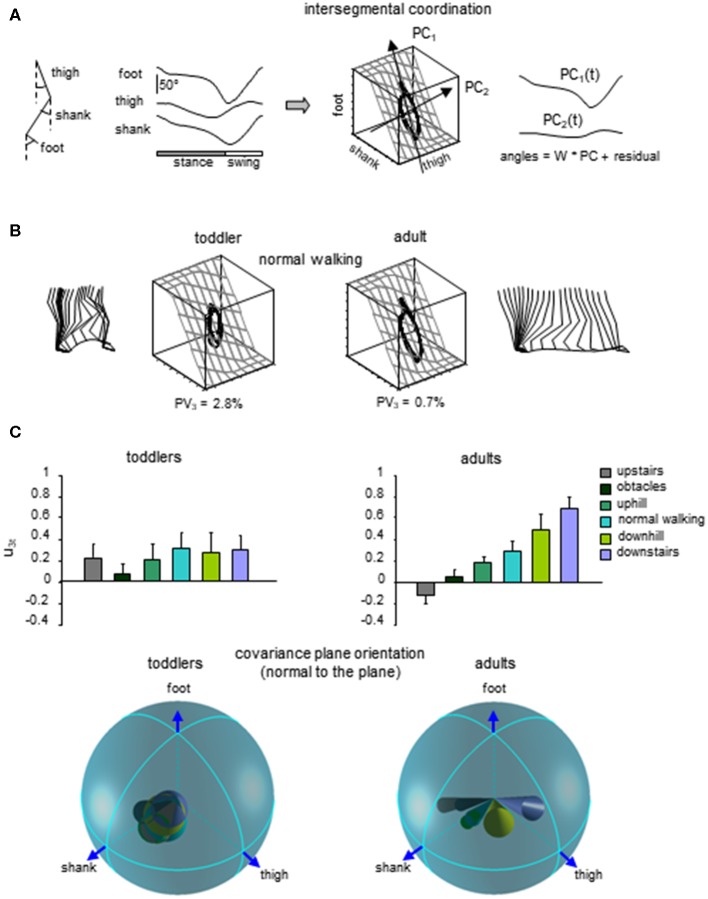
Lack of adaptability of the intersegmental coordination to different locomotor conditions in toddlers. **(A)** intersegmental coordination assessed by principal component analysis (PCA) of limb segment elevation angles during walking. From left to right: thigh, shank, and foot elevation angles (relative to the vertical), corresponding 3D trajectory in segment angle space along with the interpolated plane and the directions of PC_1_ and PC_2_, and two principal components that account for ~99% of variance of three elevation angles in adults (modified from Ivanenko et al., 2008b). **(B)** examples of gait loops and interpolation planes during walking in one toddler and one adult. Stick diagrams for one stride are also shown. Percent of variance explained by the third PC (PV_3_) is indicated, and reflects the deviation from planarity. **(C)** changes in the orientation of the covariance plane during walking over different surfaces in adults and a lack of these adaptations in toddlers. Upper panels: projection of the normal to the covariance plane onto the thigh axis (u_3t_, mean + SD). Lower panels: spatial distribution of the normal to the plane (u_3_, the angles of cones correspond to 2 spherical angular dispersions) for each condition (modified from Dominici et al., 2010).

Throughout the development, a progressively greater occurrence of initial heel contacts is observed during walking (Figure 4B) (Bertsch et al., 2004; Hallemans et al., 2006b), along with maturation of the control of foot trajectory (Forssberg, 1985; Dominici et al., 2007) and intersegmental coordination (Figure 5B) Cheron et al., 2001; Ivanenko et al., 2004; Dominici et al., 2011. Meanwhile, the timing and amplitude of muscle activities are gradually tuned to the mechanical behavior (Okamoto et al., 2003; Dominici et al., 2011; Teulier et al., 2012; Sylos-Labini, La Scaleia, Cappellini, Fabiano, Picone, Keshishian, 2020; Cheung et al. under review). The muscle activations are progressively shaped to produce the desynchronized joint extension, and the lumbar and sacral loci of activation become more dissociated (Figure 3) with shorter activation durations (Ivanenko et al., 2013a; Cappellini et al., 2016).

The progressive emergence of mature gait suggests that these patterns result from the neural maturation of central pathways, as well as a better integration of central commands with sensory signals (Yang et al., 1998). In adults, various cerebral cortices are involved in the control of locomotion (Leyton and Sherrington, 1917; Drew, 1988; Fukuyama et al., 1997), with some of them predominantly participating in the control of running rather than walking (Suzuki et al., 2004; Jahn et al., 2008). Neonates have most likely weak descending input (Yang and Gorassini, 2006). Indeed, many structures of the central nervous system are not mature at birth. For example, corticospinal tract axons become progressively myelinated only during the first 2–3 years of life (Richardson, 1982; Brody et al., 1987; Kinney and Volpe, 2018). While the involvement and the functionality of supraspinal structures for gait control have been little investigated in infants (see however Petersen et al., 2010), one can hypothesize that features of mature gaits are progressively added with the maturation and the gradual integration of supraspinal, intraspinal, and sensory control. In summary, the collective variables of mature patterns are not fully implemented at birth (Figures 3, 4, 5B), raising questions about complete innateness of gait-specific circuitry differentiation (Grillner and Wallén, 2004).

## Lack of Gait Transition in Infants

**Figure 6:**
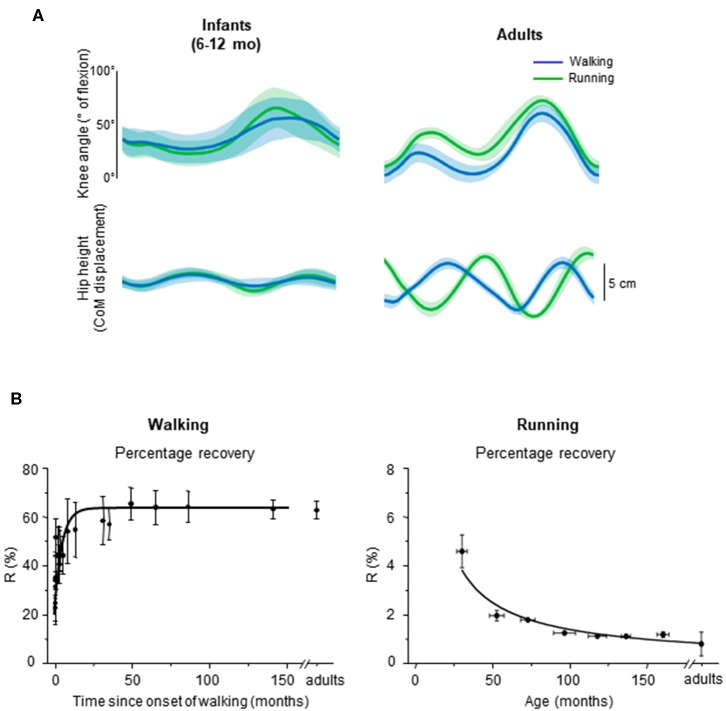
Emergence of walking and running gait features during development. **(A)** comparison between knee angle (top) and hip vertical trajectory (bottom) in infants and adults, adapted from Vasudevan et al. (2016). Walking (blue) and running (green) gaits were defined based on the presence of double support period or flight phase, respectively. Note similar kinematic patterns in infants and distinct patterns in adults for both gaits. **(B)** percentage of recovery of mechanical energy (*R*%) during walking as a function of the time after the onset of independent walking (left) (adapted from Ivanenko et al., 2004), and *R*% during running as a function of age (data from Schepens et al., 1998). During walking, a greater *R*% reflects a potentially better pendular energy exchange whereas during running, a smaller *R*% reflects a potentially better elastic energy exchange.

Another evidence for the lack of differentiation between the two gaits in early infancy is the absence of clear gait transition events. Indeed, an essential criterion for gait distinction has been formulated by defining a gait as “a pattern of locomotion characteristic of a limited range of speeds described by quantities of which one or more change discontinuously at transitions to other gaits” (Alexander, 1989). Adults spontaneously walk at slow speeds and run at faster speeds, so that transitions from one gait to another generally occur when speeding up or slowing down and when one gait mode tends to become energetically more efficient than the other one. Despite small variations depending on walking conditions (De Smet et al., 2009; Van Caekenberghe et al., 2010), a spontaneous transition from walk to run occurs around 2 m/s (Van Caekenberghe et al., 2010; Ganley et al., 2011; Segers et al., 2013) and is typically abrupt (Raynor et al., 2002; Segers et al., 2013). In particular, the gait transition is marked by a discontinuous change in intralimb coordination (Saibene and Minetti, 2003; Ivanenko et al., 2011). This modification of coordination is related to the shift from the relatively straight leg of walking to the compliant spring leg behavior of running. This difference in support leg length during stance is clearly reflected by the trajectory of the hip and the knee joint angle (Figure 6A, right panels).

In infants, Vasudevan et al. (2016) analyzed the hip trajectory and knee joint angle during supported stepping on a treadmill in a large range of speeds (from 0.06 to 2.36 m/s; i.e., even above adult spontaneous walk to run transition). As speed increased, a period of flight started appearing, which suggests that infants switched from walk to run. However, when comparing the hip trajectory and knee joint angular motion during gait without (slow speeds) and with (fast speeds) flight phases, the authors did not find an altered intralimb coordination or a modified hip trajectory in a manner that would suggest adult-like gait transition (Figure 6A, left panels).

It is unlikely that the smoothness of gait transitions and the lack of differentiation between gaits are attributed to experimental conditions, such as partial body weight support in infants. In adults, a lack of abrupt changes was observed with a simulated lower level of gravity (Ivanenko et al., 2011; Sylos-Labini et al., 2011), supporting the idea that loading conditions may be a major trigger of the transition to running (Segers et al., 2013). To record infant stepping on a treadmill (Vasudevan et al., 2016), infants were manually supported (~55% of their weight were supported, on average). Therefore, these authors also compared adults with 50% body weight support and observed that changes in intralimb coordination at the walk-to-run transition in adults remained. While body weight support may affect the abruptness of the walk-to-run transition, it does not completely eliminate signs of gait transition. The fact that the infants do not display distinct intralimb coordination and COM trajectory across a large range of speeds (Figure 6A) suggests that the gait-related neural circuitries are not mature yet.

## Biomechanical Factors and Development

In addition to above-mentioned maturation of the neural circuitries, biomechanical factors also play a role in locomotor development (Thelen, 1995; Adolph and Robinson, 2015; Adolph et al., 2018). In humans as in other animals, locomotion behavior in its different forms arises from the closed-loop interaction between the neural output, the physical dynamics of the mechanical system (inertia, viscoelastic properties of muscles and tendons and body size) and the ability to adjust the movement to the external environment (Taga, 1995; Hatsopoulos, 1996; Aoi and Tsuchiya, 2006).

First, some influences on gait patterns in infants might be expected due to differences in the anthropometry, shape, and mass distribution across different body segments, all parameters changing considerably during the entire course of development through adult age. This implies a continuous update of the neural commands to take into account the changing mechanical factors. Different mass distribution across body segments in infants and, in turn, the location of the COM, induces modifications of balance (Druelle et al., 2016), which potentially affects the emerging locomotor behavior (Clark et al., 1988). The shape of the body, bone morphology (Shefelbine et al., 2002; Cowgill et al., 2010; Raichlen et al., 2015), and foot structure (Maier, 1961; Bosch et al., 2007; Price et al., 2018) in infants are different from adults, and they also change with limb loading and locomotor experience during development. In particular, a child's foot goes through significant developmental changes in shape and soft tissues of the foot sole, e.g., the presence of a fat pad underneath the foot plantar surface in infants and slow ossification of intrinsic foot bones during the first years after birth (Maier, 1961; Gould et al., 1989; Bertsch et al., 2004). These latter factors are especially important since the spring-like longitudinal arch is a unique feature of the human foot, an essential evolutionary adaptation to the “bouncing” mechanism of running (Holowka and Lieberman, 2018; Venkadesan, Yawar, Eng, Dias, Singh, Tommasini, 2020). Therefore, different foot functions might be expected in infants as compared to spring-like behavior of the adult foot (Wager and Challis, 2016; Kelly et al., 2018).

Second, there might also be important peripheral contributors to the lack of adult-like locomotor patterns in early infancy, in particular due to slower and weaker muscles. For instance, based on the principle of dynamically similar locomotion (taking into account the differences in body proportions by comparing adults and infants moving at the same Froude number, Cavagna et al., 1983; Alexander, 1989; Schepens et al., 2004, neonates would be expected to show walk-to-run transition at ~2 km/h (since their limb is about 6 times shorter), which would correspond to ~0.25 s stride duration. Significantly wider muscle activation bursts in infants (Dominici et al., 2011; Cappellini et al., 2016; Sylos-Labini, La Scaleia, Cappellini, Fabiano, Picone, Keshishian, 2020) would further compromise the control of such short running cycles. Furthermore, given that skeletal muscles are substantially slower in neonates due to the absence of fast fibers at birth (Denny-Brown and Sherrington, 1929; Buller et al., 1960), it would be problematic for them to accurately control such short gait cycles. In fact, even during stepping, the stride duration in neonates (~2–5 s) is considerably longer than in adults (~1 s) (Ivanenko et al., 2013a). Even in older (11–13 yrs) children, muscle contraction time is ~50% longer than in adults (Dayanidhi et al., 2013), and running requires faster limb oscillations due to shorter limbs (Schepens et al., 1998), which might possibly explain why children also display a third gait mode—“skipping”—requiring slower limb oscillations (Minetti, 1998). In addition, muscle strength also increases during development (Bäckman et al., 1989), and the deviation from adult gaits in infancy may also be related to adaptive strategies for limiting the muscle activation demands (Hubel and Usherwood, 2015).

Finally, a lack of gait-specific circuitry differentiation might also be associated with a general lack of adaptability to biomechanical constrains in infants. A key example of the closed-loop interaction between the development of neural commands and biomechanics is the emergence of multi-segmental coordinative law (Lacquaniti et al., 1999). Such a kinematic covariation between limb segment rotations has been uncovered in human walking and running (Borghese et al., 1996; Bianchi et al., 1998; Ivanenko et al., 2007a). Each segment oscillates back and forth relative to the vertical with a similar waveform, time-shifted across different segments (Figure 5A). The lower limb segment angles do not evolve independently of each other, but they are tightly coupled (Borghese et al., 1996). Indeed, when the angles are plotted one vs. the others, they co-vary along a plane, describing a characteristic loop over each stride (Figure 5A). The specific shape and orientation of the plane reflects the phase relationship between segments and therefore the timing of the intersegmental coordination (Barliya et al., 2009). Such coordination of limb segments can be described by statistical methods using principal component analysis (PCA). The two first principal components (PC1 & PC2) lie on the plane of angular covariation and describe the global form of the gait loop, whereas the third one (PC3) is orthogonal to the plane (Borghese et al., 1996). The percentage of variance accounted for by PC1 and PC2 reflect the shape of the gait loop, whereas the variance accounted for by PC3 reflects the planarity of the loop. At the onset of unsupported walking, a significant deviation from planarity is observed for the child (Cheron et al., 2001). Also, the gait loop was less elongated than in adults, and the variance accounted by PC1 (Ivanenko et al., 2004) was smaller than in adults, most likely due to a higher foot lift during swing phase (Dominici et al., 2007; Ivanenko et al., 2007a). Even if the intersegmental coordination in toddlers rapidly evolves toward the adult shape and planarity with experience (Cheron et al., 2001; Ivanenko et al., 2004; Dominici et al., 2011), when toddlers step on different support surface, they do not adapt their intersegmental coordination as adults do. Instead, they keep constant phase relationships (Figure 5C, Dominici et al., 2010). Since the changes in planar covariation are thought to reflect the ability to adapt to different gait conditions (Bianchi et al., 1998; Martino et al., 2014; Dewolf et al., 2018), such as walking and running (Ivanenko et al., 2007a), the lack of changes observed in toddlers suggest a reduced flexibility of gait (Dominici et al., 2010), and potentially the absence of distinct gait patterns at the onset of independent locomotion.

## Emergence of Different Modes of Locomotion During Growth

“*During the second year of life, toddler's locomotion is neither walking, nor running, but something not yet differentiated”*

Bernstein (1947)

First emphasized by Bernstein (1947), the above-considered observations are consistent with the idea that locomotor patterns in infants are immature and lack adaptive features. Indeed, with walking experience, the gait-specific collective variables progressively become close to the values obtained in adults (Whitall and Getchell, 1995). For instance, despite millions of years of bipedal evolution, at the onset of independent walking the pendulum-like mechanism of walking and the bouncing mechanism of running deviate significantly from those of adults (Figure 6B) (Ivanenko et al., 2004, 2007b; Legramandi et al., 2013). During running, a flight phase progressively occurs and increases as the vertical push provided by muscles increases with age (Legramandi et al., 2013). These changes with aging are concomitant with an enhancing of the elastic bounce that characterizes adult running gait (Schepens et al., 1998), as the percentage of pendular COM energy exchange decreases (Figure 6B). During walking, toddlers at the onset of unsupported locomotion fail to demonstrate a prominent pendular energy exchange (Cheron et al., 2001; Ivanenko et al., 2004, 2007b) as well as an adult-like heel-to-toe roll-over pattern (Figure 4). With walking experience, the hip trajectory and the pendulum-like exchange of energy progressively evolve toward mature values (Figure 6B) (Ivanenko et al., 2004; Schepens et al., 2004; Van de Walle et al., 2010). At the same time, the foot-contact pattern shows a trend from initial forefoot to initial heel contact (Figure 4C), and the vertical force waveform slowly evolves toward a double-hump shape (Figure 3) (Hallemans et al., 2006b).

The gradual evolution of gaits after the onset of independent locomotion supports the idea that the original spinal networks are still used, but that gait-specific neural circuits mature progressively during development. Interestingly, the modification of intralimb coordination, vertical force, and spinal motor pools during walking in elderly adults suggests that aging causes a regression of the locomotor pattern: the ability to adapt the intersegmental coordination to speeds is reduced (Dewolf et al., 2019b), the second apex of the m-pattern progressively decreases (Meurisse et al., 2019), while the burst of sacral motor pools occurs earlier during the step cycle (Monaco et al., 2010). Even if far less attention has been devoted to the development of running skills, running most likely evolves from the same original spinal networks, and also requires maturation and experience. During growth, the older the child, the closer the waveform to the adult. It is interesting to note that, as in walking, these trends slowly reverse during the course of the life (Cavagna et al., 2008).

While the involvement of gait-dependent spinal interneurons has been emphasized above, the lack of evidence for distinct walking and running patterns in infants and the progressive developmental bifurcation between the different forms of gait suggest a sharing of circuitry before the full maturation of the brain and its descending inputs. In humans, the maturation of walking corresponds to maintenance of primitive patterns with superimposition of additional patterns (Dominici et al., 2011; Sylos-Labini, La Scaleia, Cappellini, Fabiano, Picone, Keshishian, 2020), and the maturation of running may also involve fine-tuning and reshaping of these primitive patterns (Cheung et al. under review). In adults, despite diverse biomechanical demands of running and walking, few patterns of muscle activation are also shared (Cappellini et al., 2006; Yokoyama et al., 2016), indirectly supporting a common neural origin for the two gait forms in infancy.

Such maturation is probably a process in which environmental signals act to bring about the characteristics of adult-like walking (Forssberg, 1992). This is supported by the fact that independent stepping acts as a functional trigger of gait maturation (Ivanenko et al., 2004; Yang et al., 2015), and Earth's gravity has a significant impact on early development of motor functions (Cheron et al., 2001; Ivanenko et al., 2007b). It is indeed well-documented that the interaction with the environment influences the development of motor networks. For example, early exposure of animals to altered gravitational field (hypo- or hyper-gravity) affects their mature motor performance (Sondag et al., 1997; Walton, 1998; Wubbels and de Jong, 2000; Walton et al., 2005; Bojados et al., 2013). Of particular interest, hyper-gravity reduces the postnatal development of descending inputs to the spinal cord (Brocard et al., 2003), suggesting that gravity has a critical role to shape the maturation of gait-specific pattern generation circuitries. When adult humans are exposed to microgravity, they start to rely more on skipping (a potential vestigium of gallop) or running gait (Pavei et al., 2015; Lacquaniti et al., 2017). It is therefore plausible that, if humans were even born on the Moon, modification of the chronology of the emergence of gait during development (and even novel locomotor behaviors) would occur, starting from the same inborn motor primitives.

The interactions with the environment that shape the emergence of gait is conceivable, because stepping development in infants highlights strong plasticity. For example, Patrick et al. (2014) showed that the interlimb coordination can be manipulated with a 4-week training, indicating experience-dependent learning at a young age (<10 months). In animals, early motor experience influences the muscle typology (Serradj and Jamon, 2016). Such impact of training procedures suggests that experience is required for normal development of locomotor behavior and that motor output in adults could be optimized by appropriate training during a defined period of motor development (Walton et al., 1992; Muir and Chu, 2002; Serradj and Jamon, 2016). Accordingly, human infants undergoing daily stepping exercise exhibit an earlier onset of independent walk than untrained infants (Zelazo et al., 1972; Yang et al., 1998).

As in animals, it is plausible that the two modes of locomotion and their corresponding neural circuits have different maturation rate. While the running pattern of chicks seems mature earlier in life (Muir et al., 1996), the current coarse picture of the development of running patterns in infants needs to be refined at different developmental stages, providing important insights into the process of skills acquisition. Even if the patterns of innate stepping differs from the efficient running adult gait, several parameters bear a striking resemblance to the mature running patterns (Figures 3, 4C), such as the vertical force, the motoneuron activity of the lumbosacral enlargement, diverse foot contact strategies and knee flexed throughout stance (Yang et al., 2015; Vasudevan et al., 2016; Sylos-Labini et al., 2017). A hypothetical innateness of some features characteristic of adult running is also compatible with the evaluation of the evolution of the human body form. Indeed, Rolian et al. (2009) suggest that the modern human forefoot proportions might be part of a suite of adaptations selected especially for running gait that evolved in the genus Homo around 2 million years ago (Bramble and Lieberman, 2004; Venkadesan, Yawar, Eng, Dias, Singh, Tommasini, 2020). Hypotheses of innate behavior should always be taken in a relative terms, since any behavior is modified by experience (Grillner and Wallén, 2004; Vanden Hole et al., 2017).

## Concluding Remarks

The emergence of adult-like walking and running patterns results from the evolution of multiple subsystems of the developing child, involving both neural and biomechanical factors (Thelen and Ulrich, 1991). While the locomotor output of stepping neonates has been widely compared to the adult walking, its comparison with adult running patterns needs to be explored further to unravel some of the mysteries surrounding the progressive bifurcation of the locomotor networks at different developmental stages. The delayed onset of running in children may be related to environmental and musculoskeletal factors and a limited ability to adapt to biomechanical constrains in infants (Thelen, 1995). The findings we reviewed in this article point to a partial overlap in the neural and biomechanical control of walking and running in infancy, suggesting that different forms of gait are built starting from common, likely phylogenetically conserved elements.

Gaining insights into the maturation and differentiation of human gaits may also provide important clinical implications. For instance, while the rehabilitative protocols in children with cerebral palsy usually focus on walking training, early running training may also be beneficial, and improve walking gait (Lewis, 2017). Indeed, many studies have examined how children with cerebral palsy manage to walk, but few have investigated running, which may be even more stable than walking in these children (Iosa et al., 2013). There may also be critical developmental windows during which specific experiences have a greater effect on the early developmental process and differentiation of locomotor behaviors than at other times (Ivanenko et al., 2013b; Yang et al., 2013). Taking advantage of newly available biotechnological approaches and techniques (Zhu et al., 2015; Chung et al., 2019; Redd et al., 2019; Xu et al., 2019; Airaksinen et al., 2020) for both advanced neurophysiological pediatric recordings and rehabilitation training in the sensitive period for maturation (e.g., using biofeedback or neuromodulation) would help to diagnose and assess early motor deficits and to determine the activity-based intervention for infants with developmental disorders.

## Author Contributions

All authors listed have made a substantial, direct and intellectual contribution to the work, and approved it for publication.

## Conflict of Interest

The authors declare that the research was conducted in the absence of any commercial or financial relationships that could be construed as a potential conflict of interest.
